# Surprisal Analysis-Based Compaction of Entangled Molecular States of Maximal Entropy

**DOI:** 10.3390/e28020192

**Published:** 2026-02-09

**Authors:** James R. Hamilton, Francoise Remacle, Raphael D. Levine

**Affiliations:** 1 The Fritz Haber Center for Molecular Dynamics and Institute of Chemistry, The Hebrew University of Jerusalem, Jerusalem 91904, Israel; jamesross.hamilton@mail.huji.ac.il (J.R.H.); fremacle@uliege.be (F.R.); 2 Theoretical Physical Chemistry, UR MOLSYS, University of Liège, B4000 Liège, Belgium; 3 Department of Molecular and Medical Pharmacology, David Geffen School of Medicine, University of California, Los Angeles, CA 90095, USA; 4 Department of Chemistry and Biochemistry, University of California, Los Angeles, CA 90095, USA

**Keywords:** ultrafast excitation, quantum information theory, algebraic dynamics, sudden approximation, vibronically excited N_2_

## Abstract

An attosecond optical pulse can entangle coherently related states of different characters, such as electronic and vibrational, in a molecular system. Using a quantum information theoretic approach, we explicitly define and discuss the surprisal of such a system in the maximal entropy formalism and identify the constraints and their conjugate Lagrange multipliers. Surprisal analysis shows how these constraints become fewer and simpler in the sudden approximation of the dynamics, a limit often valid for an ultrafast excitation. The optically accessible lower electronic states of N_2_ are used as a numerical example to show the compaction of the dynamics from $O\left(n^{2}\right)$ down to $O\left(n\right)$ constraints, where n is the number of vibronic states. The von Neumann entropy is used to confirm the fidelity of the compaction.

## 1. Introduction

In the late 1960s experiments in molecular reaction dynamics [1] started providing detailed population distributions of the internal states of molecules. Surprisal analysis [2] was introduced to compact and interpret these incoherent, classical-like distributions. A similar, analogous line of development unfolded in non-relativistic nuclear heavy ion collisions [3] and surprisal analysis was applied here too [4]. In both disciplines, the experimentally observed distribution of population states could be represented in terms of very few, one or two, constraints. The constraints reduce the entropy of the prior distribution that is taken as the distribution of quantum states subject only to constant, conserved observables such as energy or number of nucleons.

In quantum mechanics, a system is characterised not only by the populations of states but also by the coherences of states. In other words, one needs the density matrix [5]. It has been shown [6] that when the density matrix of a system has an initial maximal entropy form, it retains this form both during and after a collision or external perturbation. The condition on the quantum dynamics is that the time evolution operator is unitary, which is a standard result for a Hermitian Hamiltonian (see Complement C_II_ of [7] and also [8]). Considerable simplification is possible if the Hamiltonian and the constraints can all be expressed as linear combinations of the generators of a Lie algebra. This enables a great simplification, but very sadly it is only valid for systems that are multiply harmonic. Realistic systems are typically anharmonic, and their algebraic description requires Hamiltonians that are at least bilinear in the generators.

In this paper we recognise that anharmonic systems typically have only a finite number of bound states. Such systems have, of course, a continuum. However, at lower energies, for systems where one starts in the ground state or in a thermal state at ordinary temperatures, a realistic perturbation hardly accesses the continuum. The continuum can be that of the photoionization to the cation, or the dissociative continuum when the potential of the excited states is shallow. It is therefore reasonable to approximate the dynamics by restricting the set of states to the bound ones. As we show, this allows us to define a set of generators in which the unperturbed Hamiltonian and the perturbation, both of which become finite matrices, are linear in the generators.

A new highly promising and challenging experimental development is the introduction of ultrafast laser pulses [9,10], as recognised by the Nobel Prize in Physics for 2023 for the study of electron dynamics in matter [11]. The short duration and thereby wide spectral range of such pulses, a consequence of the Heisenberg time-energy uncertainty (see Complement D_XIII_ of [12] and also [13]), allows for the coherent excitation of several quantum states. In molecules one can thereby entangle [14] dynamics of different degrees of freedom [15,16,17,18,19,20,21,22,23]. A detailed example of entanglement of excited electronic states and vibrational states in N_2_ is presented in [20]. A special aspect of this paper was the compaction of the entangled wave function using the procedure of singular value decomposition. The density matrix of an entangled state exhibits coherence between different states and, as such, it is as far as one can get from a classical-like distribution of the population of quantum states. So, we here seek to show how surprisal analysis can be applied to the analysis and compaction of the quantum mechanical state. We choose the previously studied example of an attosecond-pumped molecule of N_2_ in the lowest set of optically allowed excited electronic states of pi symmetry [24,25]. We examine the quantum mechanical density matrix and seek to compact it in the framework of the maximal entropy formalism [26,27]. Explicitly we seek the smallest number of constraints on the entropy of the state that provides an exact representation. We determine that there are separate sets of constraints which govern the populations of states and the coherences, and thereby the entanglement.

As discussed, it typically requires an ultrafast excitation to generate an entangled state. Such an excitation has a very broad spectral range that spans several quantum states. These conditions make a sudden approximation of the dynamics realistic. This allows for a simplification of the compaction, as was previously shown with just the vibrational excitation in the ground electronic state of a Morse oscillator [28].

Before we turn to the technical discussion we very briefly define entanglement in our context.

Our system has both electronic and vibrational states. A general form of the molecular wave function is the linear combination $\left.|\Psi \left(t\right)\right\rangle =\sum_{i,j}a_{ij}\left(t\right)|\left.e_{i}\right\rangle |\left.v_{j}\right\rangle$ of products of electronic states $|\left.e_{i}\right\rangle$ and vibrational states $|\left.v_{j}\right\rangle .$ Entanglement means that the matrix of coefficients $\left\{a_{ij}(t)\right\}$ cannot be reduced such that only one term suffices in the linear combination. Entanglement of electrons and nuclei means that the density matrix of the system has coherences of the form $\left|\left.e_{i}\right\rangle \right|\left.v_{j}\right\rangle \left\langle v_{k}\right.|\left\langle e_{l}\right.|$.

In its original conception [29] entanglement referred to the entanglement of two particles. In chemistry this is still of interest. A recent example is the question of whether two electrons in a bound molecule are entangled [30].

This paper has three technical sections. Section 2 introduces ideas of compaction and the form of a compacted surprisal, Section 3 defines the N_2_ system and its algebraic aspects, and Section 4 reviews the sudden approximation and its application. The Lie algebraic approach to surprisal analysis in a finite dimensional Hilbert space is discussed in Section 5 and continued in Section 6. The key results about dynamics and compaction are in Section 6. We make our concluding remarks in Section 7.

## 2. About Compaction

Good compaction, that is, maximal reduction in the size of a data object with minimal loss of information, requires the exploitation of some kind of structure in the code being compressed. In the quantum information theoretic approach employed in this work, which applies information theory to molecular dynamics, the data object to be compressed is the density matrix of the quantum system. Or, more precisely, the surprisal of the density matrix in its maximal entropy form.

The maximal entropy form of the quantum mechanical density matrix at the time t is $\rho \left(t\right)=\exp\left[-I\left(t\right)\right]$, where the exponent, I, is the surprisal. It is represented as a sum of operators—referred to as constraints—$\left\{X_{k}\right\}$ and their weights, technically known as the Lagrange multipliers, $\left\{\lambda_{k}\right\}$, $I=\sum_{k=0}^{N}\lambda_{k}X_{k}$. The time dependence can be in the constraint, $\sum_{k}\lambda_{k}X_{k}\left(t\right)$, or, in favourable cases, it is the Lagrange multipliers that carry the time dependence, I = $\sum_{k}\lambda_{k}\left(t\right)X_{k}$. N is the number of constraints needed to construct the uncompacted surprisal. Compaction is the process whereby the number of constraints in the surprisal is reduced from $N\to N_{c}$. Note that the constraints and Lagrange multipliers in the compacted surprisal are not necessarily the same as those of the uncompacted surprisal, $I_{c}=\sum_{k=0}^{N_{c}}\lambda_{k}^{c}Y_{k}$. For the compaction to be significant, the order of magnitude of the number of terms should be reduced, $\ln\left[N_{c}\right]\ll ln\left[N\right]$.

Compaction can be either lossless or lossy. We use these terms in their standard information theoretic sense. Lossless compaction reduces the “size” of a data object, without any reduction in its information content as it is compressed. Lossy compaction reduces the size of the data object, but with some loss of information in the compression. Lossy compaction can still be acceptable if the amount of information lost in the compression is not significant.

In quantum information theory, the measure of information is the von-Neumann entropy, $S=-tr\left[\rho \ln\left[\rho \right]\right]$. For a dynamical state the entropy will be a time-dependent quantity, but for the avoidance of clutter, the explicit time dependence is generally dropped in this paper. The von Neumann entropy is the measure of the information one has about the quantum system. If the system is in a pure state, for example, all the population is in the ground state, the entropy of the system is zero. S=0 means one has complete information and no uncertainty about the system. One knows what state it is in. However, this is not the case if the system is in a mixed state, for example, in a thermal distribution $\rho_{0}=Z^{-1}\exp\left[-\beta H_{0}\right]$, where Z is the partition function, $\beta =1/k_{B}T$, $k_{B}$ being the Boltzmann constant and T being the temperature, and $H_{0}$ is the unperturbed Hamiltonian. Such a mixed state will have a finite entropy. This is because one has less knowledge—more uncertainty—about what state the system is in. One is maximally uncertain when the population distribution of the states is incoherent and the population distribution is uniform, i.e., all quantum states are equally probable [31,32].

When a system is compacted, the loss of information caused by the compaction is quantified as

(1) $$\begin{array}{c} \Delta S=tr\left[\rho \ln\left[\rho_{c}\right]\right]-tr\left[\rho \ln\left[\rho \right]\right],\; \end{array}$$

where $\rho =\exp\left[-I\right]$ is the uncompacted density matrix of maximal entropy and $\rho_{c}=\exp\left[-I_{c}\right]$ is the compacted. It is always the case that ΔS≥0, in other words, compaction cannot increase one’s information, or reduce one’s uncertainty, about the system. For lossless compression ΔS=0. For lossy compression ΔS>0. Lossy compression is acceptable if ΔS≪S.

Note, an alternative measure of the increased uncertainty resultant from lossy compression is the fidelity of the compacted density matrix to the original

$$F\left(\rho ,\rho_{c}\right)=tr\left(\sqrt{\sqrt{\rho }\rho_{c}\sqrt{\rho }}\right).$$

The fidelity ranges from 0 to 1. A fidelity of $F\left(\rho ,\rho_{c}\right)=1$ means the information is perfectly preserved, and $F\left(\rho ,\rho_{c}\right)=0$ means that all information is lost. We have shown elsewhere that the fidelity and entropy difference correspond [28].

## 3. The N_2_ System

### 3.1. The Gelfand Basis

The $n^{2}$ Gelfand *n* by *n* matrices $\left\{E_{ij};\;i,j=1,2,\ldots ,n\right\}$ are defined such that a matrix $E_{ij}$ has the element value one at the intersection of row i and column j and zeros elsewhere. This set of matrices is a very useful basis set of operators for representing both the unperturbed Hamiltonian, the perturbation and any Hermitian operator $\hat{A}$ that can be represented as a matrix in the basis of the *n* bound states, $\left\{|\left.i\right\rangle ;\;i=1,2,\ldots ,n\right\}$ $A=\sum_{i,j}e_{ij}\;E_{ij}\;,\;e_{ij}=\left\langle i|\hat{A}|j\right\rangle$.

The Gelfand matrices, $\left\{E_{ij}\right\},$ are known to be generators of a Lie algebra [33,34] as they are closed under commutation $\left[E_{ij},E_{kl}\right]=E_{il}\delta_{jk}-\;E_{kj}\delta_{li},$ where the delta symbol is the familiar Kronecker delta. In terms of a particular orthonormal basis set it can be helpful to write in the Dirac notation $E_{ij}=\left|\left.i\right\rangle \left\langle j\right.\right|.$

### 3.2. The Structure of the Unperturbed N_2_ Molecule

The system to be used in this work for the discussion of the dynamics of an entangled state is the ground electronic state of the nitrogen diatomic, X ΣG+1, and its first three one photon-accessible excited electronic Πu1 states, $\left\{b,c,e\right\}$.

The total number of vibrational states of a system will be the sum of the vibrational states of the constituent ground and electronic states, $v_{tot}=\sum_{j=\left\{G,E1,E2\ldots \right\}}v_{j}$, where the G and Ei in the summation signify the ground and i^th^ excited electronic states respectively. From [24,25] the number of ground electronic vibrational states is $v_{G}=82$, and the numbers of excited electronic vibrational states are $v_{b}=39,$ $v_{c}=63$ and $v_{e}=73$. The numerical examples presented in this work will not be done for the full $v_{tot}=257$ vibrational state system. To demonstrate the principles behind the methods of surprisal analysis and compaction, it is sufficient to look at a reduced number of states.

The reduced N_2_ system to be used in the numerical sections consists of a ground electronic state with a single vibrational state, and the three excited diabatic electronic pi states, a lower lying shallower valence excited state with a manifold of eight vibrational states and two tightly bound Rydberg excited states of far higher vibrational frequency, each with four vibrational states $\left(v_{tot}=17\right)$.

### 3.3. The Optically Pumped N_2_ Molecule

The reduced N_2_ $\left(v_{tot}=17\right)$ system will be pumped by an ultrashort VUV optical pulse. The carrier frequency of the optical pulse, ω, will be calibrated to approximately the energy difference between the ground and excited electronic states. As such, it will only be resonant with the ground to the electronically excited state transitions. It will not be resonant with transitions between the vibrational states or within the excited electronic states. Therefore, only the dipole moments for the ground to excited electronic states are needed. The dipole transition moments between Morse vibrational states can be computed with a useful discussion by [35] and by [36]. The electronic transition moments as a function of the N_2_ bond distance are given in [24] and observed and computed values are reported in [37].

The energies and transition dipole moments of the states in the reduced system are given in Table 1.

### 3.4. The Algebraic Structure of a System of Multi-Electronic States

The algebraic Hamiltonian for a system of multi-electronic states each with its manifold of vibrational states is here taken to be diagonal $\left\langle i|H_{0}|j\right\rangle =\delta_{ij}E_{i}$ or, in terms of the Gelfand matrices

(2) $$\begin{array}{c} H_{0}=\sum_{i}E_{i}E_{ii},\; \end{array}$$

where $E_{i}$ is the energy difference between the ground electronic, ground vibrational state and the i^th^ state. The use of the diabatic electronic states as diagonalizing the Hamiltonian is an approximation which neglects their electronic coupling. As discussed below we use a rather short pulse to excite the molecule, which will be over in a much shorter time [21] than it takes for the diabatic states to be effectively coupled.

The time dependent Hamiltonian of the system is

(3) $$\begin{array}{c} H\left(t\right)=H_{0}+V\left(t\right)\; \end{array}$$

where the time dependent perturbation term is

(4) $$\begin{array}{c} V\left(t\right)=-E\left(t\right)\sum_{ij}\mu_{ij}E_{ij}.\; \end{array}$$

The elements of μ, the dipole transition matrix, are indexed as shown in Table 2. $\mu_{ij}$ is the transition dipole between the $i^{th}$ state and the $j^{th}$ state. Note that for the VUV pulse used here, we only need to include the transition dipoles connecting the ground state to the different excited states. Hence, in $\mu_{ij}\neq 0$ for i≠1.

$E\left(t\right)$ in Equation (4) the optical pulse used to perturb the system

(5) $$\begin{array}{c} E\left(t\right)=E_{0}\exp\left[-\frac{{\left(t-t_{0}\right)}^{2}}{2\sigma^{2}}\right]\left(\cos\left[\omega t\right]-\left(\frac{\left(t-t_{0}\right)}{\omega \sigma^{2}}\right)\sin\left[\omega t\right]\;\right),\; \end{array}$$

where $E_{0}$ is the electric field strength, σ is the duration of the pulse (the Gaussian full width, half maximum FWHM $=2\sqrt{2ln2}\sigma =2.35\sigma$), ω is the carrier frequency, and the time $t_{0}$ is centre of the Gaussian envelope.

## 4. The Sudden Approximation

When a diatomic or polyatomic molecule is perturbed by a force or pulse with sufficiently short duration, the perturbation will be over before the atoms have time to respond and transfer energy vibrationally. When such a sufficiently fast perturbation is used, the sudden approximation of the Hamiltonian can be valid. In our multi-electronic state system, even if the perturbation is sudden with respect to the vibrational energy difference, it need not be sudden with respect to the electronic energies $\left\{T_{i}\right\}$, where $T_{i}$ is the energy of the i^th^ excited electronic state.

The sudden approximation of the Hamiltonian (Equation (3)) used to model the diatomic molecule is

(6) $$\begin{array}{c} H^{s}\left(t\right)=\sum_{i}T_{i}E_{i,i}+V\left(t\right) \end{array}$$

To discuss the sudden approximation in our multi-electronic state system we write the total rate of change in the perturbation as a Heisenberg equation of motion

$$dV(t)/dt=i\partial V(t)/\partial t+\left[V\left(t\right),H_{0}\right].$$

Using the Hamiltonian of Equation (6), written below explicitly as Equation (7), it follows that the duration of the pulse needs to be smaller not just than the spacing of adjacent states but smaller than the widest spacing of states that are included in a given manifold. This already leads us to expect that the approximation will work better for the electronically excited valence state than for the Rydberg states.

The electronic ground state of N_2_, X ΣG+1, and the two Rydberg excited states, c Πu1 and e Πu1, have comparable, typically short periods of about 15 fs. The sudden approximation is thus valid for N_2_ when the duration of the perturbation in time is far shorter than this.

The validity of the sudden approximation will be tested on the reduced N_2_ system. As discussed and shown in Table 1, the numerical work in this paper on N_2_ will only take the first vibrational state of the ground electronic state, X ΣG+1, and a reduced number of vibrational states of the excited electronic states b Πu1, c Πu1 and e Πu1 ($v_{i}=8,\;4$ and 4, respectively). In the sudden approximation the Hamiltonian is a sum of two 17 by 17 matrices (Equation (7)). As has been said, a carrier frequency will be chosen for the pulse which is only resonant with the energy differences between the ground and excited electronic states. Therefore, one only needs to include the dipole moments for these ground-to-excited-vibrational excitation transitions in the dipole matrix, μ.

(7) $$H^{s}\left(t\right)=\left(\begin{array}{cccccccccccc} 0 & 0 & \vdots & 0 & 0 & 0 & 0 & 0 & 0 & 0 & 0 & 0\\ 0 & T_{b} & \vdots & 0 & 0 & 0 & 0 & 0 & 0 & 0 & 0 & 0\\ \ldots & \ldots & \ddots & \ldots & \ldots & \ldots & \ldots & \ldots & \ldots & \ldots & \ldots & \ldots \\ 0 & 0 & \vdots & T_{b} & 0 & 0 & 0 & 0 & 0 & 0 & 0 & 0\\ 0 & 0 & \vdots & 0 & T_{c} & 0 & 0 & 0 & 0 & 0 & 0 & 0\\ 0 & 0 & \vdots & 0 & 0 & T_{c} & 0 & 0 & 0 & 0 & 0 & 0\\ 0 & 0 & \vdots & 0 & 0 & 0 & T_{c} & 0 & 0 & 0 & 0 & 0\\ 0 & 0 & \vdots & 0 & 0 & 0 & 0 & T_{c} & 0 & 0 & 0 & 0\\ 0 & 0 & \vdots & 0 & 0 & 0 & 0 & 0 & T_{e} & 0 & 0 & 0\\ 0 & 0 & \vdots & 0 & 0 & 0 & 0 & 0 & 0 & T_{e} & 0 & 0\\ 0 & 0 & \vdots & 0 & 0 & 0 & 0 & 0 & 0 & 0 & T_{e} & 0\\ 0 & 0 & \vdots & 0 & 0 & 0 & 0 & 0 & 0 & 0 & 0 & T_{e} \end{array}\right)\;\;\;\;\;\;\;\;\;\;\;\;\;\;\;\;\;\;\;\;\;\;\;-E\left(t\right)\left(\begin{array}{ccccccc} 0 & \mu_{1,2} & \mu_{1,3} & \mu_{1,4} & \ldots & \mu_{1,16} & \mu_{1,17}\\ \mu_{1,2} & 0 & 0 & 0 & \ldots & 0 & 0\\ \mu_{1,3} & 0 & 0 & 0 & \ldots & 0 & 0\\ \mu_{1,4} & 0 & 0 & 0 & \ldots & 0 & 0\\ \vdots & \vdots & \vdots & \vdots & \ldots & \vdots & \vdots \\ \mu_{1,16} & 0 & 0 & 0 & \ldots & 0 & 0\\ \mu_{1,17} & 0 & 0 & 0 & \ldots & 0 & 0 \end{array}\right),$$

where $T_{i}$ is the energy of the electronic state iΠu1, defined here as the energy of its lowest vibrational state. The full quantum dynamics are evolved numerically by integrating the Liouville–von Neumann equation for the density matrix, $i\partial \rho /\partial t=\left[H,\rho \right]$, using the full Hamiltonian of the reduced system. The full Hamiltonian is similar to Equation (7) except that, rather than the $T_{i},$ it is the vibronic energies from Table 1 that appear along the diagonal (Equation (2)). The same equation of motion is used to propagate in time a sudden density matrix, $i\partial \rho^{s}/\partial t=\left[H^{s},\rho^{s}\right]$, where $H^{s}$ is the Hamiltonian in the sudden approximation (Equation (7)). The initial state for either equation of motion is taken to be a thermal state. At any reasonable temperature it means that the entire population is in the ground electronic state.

**Figure 2:**
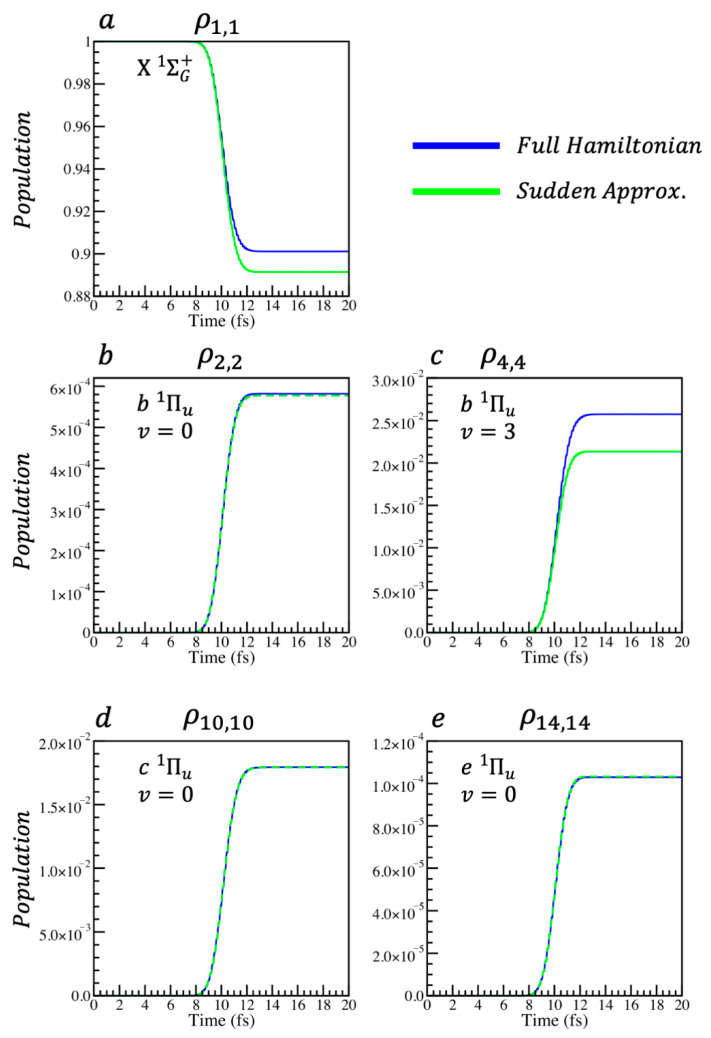
Comparison of the population elements of the reduced N_2_ system density matrices, ρ and $\rho^{s}$. (**a**) compares the ground electronic state elements. (**b**,**c**) compare respectively the v=0 and v=3 vibrational state populations of b Πu1. (**d**,**e**) compare the v=0 states of c Πu1 and e Πu1, respectively.

The following optical pulse parameters, which will be used in the pulse (Equation (5)) which drives the dynamics, are $\omega =12.75\;\mathrm{eV}=0.4687\;a.u\;and\;E_{0}=10^{-4}\;a.u.$ The central time of the pulse is at $t_{0}≅10fs=400\;a.u.$; the duration of the pulse is σ=1fs (in the time domain, the Gaussian FWHM =2.35 fs; in the energy domain, the Gaussian FWHM =1.55 eV). We chose the pulse strength $E_{0}$ to be well below any extensive population transfer to the continuum (see Figure 2).

The value of ω was chosen to be resonant with the ground to higher electronic state transition energies, but not intra-electronic state vibrational transition energies or the transition energies between excited electronic states. It is also low and narrow enough in energy so that there is no photoionization and the dynamics is restricted to bound states of the neutral N_2_.

**Figure 1:**
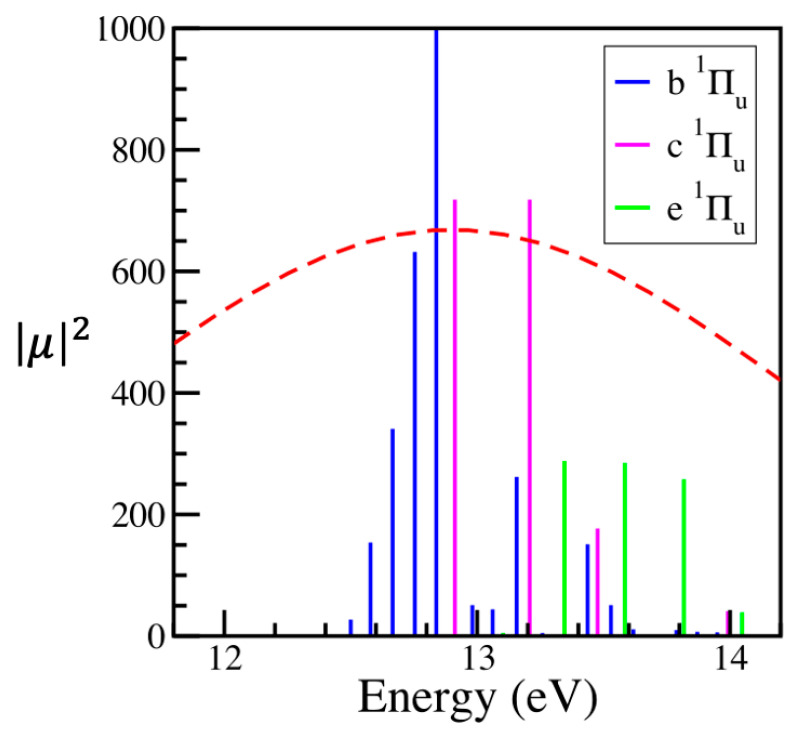
Stick spectrum of the vibrational states of the Πu1 electronic states of N_2_. The red dashed line superimposed onto the stick spectrum is the absolute value of the FT of the pulse used to excite the system. The electronic states are identified in the inset. The heights are the (squared) Franck Condon factors.

One can look at the pulse in the frequency or energy domain by taking a Fourier transform (FT). Figure 1 compares this function to the stick spectrum of the excited vibronic states of N_2_.

Figure 1 offers another way of understanding the validity of the sudden approximation. The shorter a pulse in the time domain is, the broader it will be in the energy domain. A broader envelope in the energy domain means that vibrational states which have a close energy difference from the ground state will be resonant with the pulse to a similar degree. Hence, the energy differences between vibrational states will be of limited importance when the envelope of the perturbation is broad in the energy domain. The broad pulse also means that several vibrational states can be populated; the width of the perturbation σ (the FWHM of a Gaussian pulse is 2.35 σ) is shorter than this, particularly so for the b state.

The pulse used to perturb the system is Equation (5), with the parameters given above. Hence, the comparison is of the dynamics of a perturbation within the sudden limit. Figure 2 compares the population elements of ρ and $\rho^{s}$, which have been propagated using the Liouville–von Neumann equation for the full and the sudden Hamiltonians.

## 5. Surprisal Analysis

Effective compression methods exploit the structure of the data object. In this case, the method of compression will use the matrix structure of the density matrix of maximal entropy and its surprisal. The density matrix of an entangled system has the following structure:

(8) $$\begin{array}{c} \rho =\left(\begin{array}{ccccc} \left[G↔G\right] & \left[G↔E1\right] & \left[G↔E2\right] & \left[G↔E3\right] & \ldots \\ \vdots & \left[E1↔E1\right] & \left[E1↔E2\right] & \left[E1↔E3\right] & \ldots \\ \vdots & \vdots & \left[E2↔E2\right] & \left[E2↔E3\right] & \ldots \\ \vdots & \vdots & \vdots & \left[E3↔E3\right] & \ldots \\ \vdots & \vdots & \vdots & \vdots & \ddots \end{array}\right). \end{array}$$

where G represents the ground electronic state, and Ei signifies the i^th^ excited state. $I=-\ln\left[\rho \right]$ shares this structure with ρ. Each block, $\left[i↔j\right],$ corresponds to the coherences between the vibrational states of i with those of j. There are also the population elements, $\rho_{m,m}$, which are not in these blocks, which are treated separately.

This section will begin with a general discussion of a generic entangled system and then will move on to the specific reduced N_2_ system introduced previously. There are two kinds of blocks in Equation (8). The first kind are the inter-electronic state blocks, the $\left[i↔j\right]$ with i≠j, which contain the coherences between the vibrational states of different electronic states. These blocks are the off-diagonal blocks of Equation (8), and they have the structure

(9) $$\begin{array}{c} \left[i↔j\right]=\left(\begin{array}{ccccc} b_{1,1} & b_{1,2} & \ldots & b_{1,v_{j}-1} & b_{1,v_{j}}\\ b_{2,1} & b_{2,2} & \ldots & b_{2,v_{j}-1} & b_{2,v_{j}}\\ \vdots & \vdots & \ddots & \vdots & \vdots \\ b_{v_{i}-1,1} & b_{v_{i}-1,2} & \ldots & b_{v_{i}-1,v_{j}-1} & b_{v_{i}-1,v_{j}}\\ b_{v_{i},1} & b_{v_{i},2} & \ldots & b_{v_{i},v_{j}-1} & b_{v_{i},v_{j}} \end{array}\right).\; \end{array}$$

The dimensions of the $\left[i↔j\right]$ blocks are $v_{i}\times v_{j}$, where $v_{i}$ is the number of vibrational states of electronic state i, and $v_{j}$ is the number of vibrational states of electronic state j.

The second kind of block in Equation (8) are the intra-electronic state blocks, the $\left[i↔i\right]$, which contain the coherences between the vibrational states within the electronic state i. The intra-electronic state blocks are the diagonal blocks, which have the following structure:

(10) $$\begin{array}{c} \left[i↔i\right]=\left(\begin{array}{ccccccc} 0 & c_{1,2} & c_{1,3} & c_{1,4} & c_{1,5} & c_{1,6} & ⋰\\ c_{2,1} & 0 & c_{2,3} & c_{2,4} & c_{2,5} & ⋰ & c_{v_{i}-5,v_{i}}\\ c_{3,1} & c_{3,2} & 0 & c_{3,4} & ⋰ & c_{v_{i}-4,v_{i}-1} & c_{v_{i}-4,v_{i}}\\ c_{4,1} & c_{4,2} & c_{4,3} & ⋰ & c_{v_{i}-3,v_{i}-2} & c_{v_{i}-3,v_{i}-1} & c_{v_{i}-3,v_{i}}\\ c_{5,1} & c_{5,2} & ⋰ & c_{v_{i}-2,v_{i}-3} & 0 & c_{v_{i}-2,v_{i}-1} & c_{v_{i}-2,v_{i}}\\ c_{6,1} & ⋰ & c_{v_{i}-1,v_{i}-4} & c_{v_{i}-1,v_{i}-3} & c_{v_{i}-1,v_{i}-2} & 0 & c_{v_{i}-1,v_{i}}\\ ⋰ & c_{v_{i},v_{i}-5} & c_{v_{i},v_{i}-4} & c_{v_{i},v_{i}-3} & c_{v_{i},v_{i}-2} & c_{v_{i},v_{i}-1} & 0 \end{array}\right). \end{array}$$

The dimensions of the $\left[i↔i\right]$ blocks are $v_{i}\times v_{i}$, where $v_{i}$ is the number of vibrational states of electronic state i. The diagonal elements of $\left[i↔i\right]$, which are also the diagonal elements of the ρ or $\ln\left[\rho \right]$, are zero. These $\rho_{m,m}$ are populations, and as such are not included in the coherence blocks. In Equations (9) and (10), the elements are time-dependent: $b_{m,n}\left(t\right)$ and $c_{m,n}\left(t\right)$.

Within the sudden approximation, the energies of the specific vibrational states of a particular electronic state are degenerate. The only energies in the sudden approximation of the Hamiltonian are the electronic state energies, the $T_{i}.$ Therefore, the only energy differences which appear in the Liouville–von Neumann equation, which propagates the density matrix, are the energy differences between electronic states. Of course, the energy differences do also exist in the initial $\rho_{0}$ and $\ln\left[\rho_{0}\right]$, but as they are not present in the sudden approximation Hamiltonian, they do not affect the dynamics. The only differences that the Liouville–von Neumann equation “sees” between the vibrational states of a given electronic state in the sudden Hamiltonian are their differing dipole transition moments.

**Figure 3:**
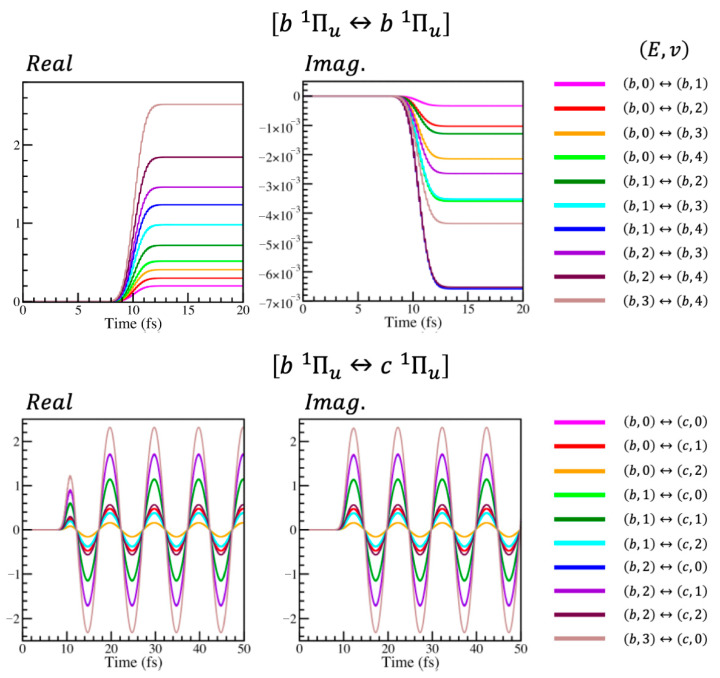
Elementwise comparison of the intra-electronic state b Πu1↔b Πu1 (**top row**) and inter-electronic state b Πu1↔c Πu1 (**bottom row**) blocks of the sudden approximation surprisal of the reduced N_2_ system. The left- and right-hand panels give the real and imaginary parts of the elements respectively. On the far left of the rows are the keys, indicating to which coherences the lines in the panels correspond; the parenthesis notation signifies the electronic E and vibrational state v as $\left(E,v\right)$.

As has been said, the energy differences between vibrational states within a specific electronic state are not present in the sudden approximation of the Hamiltonian, only the energy differences between the electronic states themselves. Therefore, the intra-electronic elements, the elements within any $\left[i↔i\right]$ block, are non-oscillatory (the top row of Figure 3). The inter-electronic elements, those within $\left[i↔j\right]$ blocks for i≠j, are oscillatory, but they all oscillate with the same frequency corresponding to the energy difference of $\left|T_{i}-T_{j}\right|$ between electronic states i and j (the bottom row of Figure 3).

**Figure 4:**
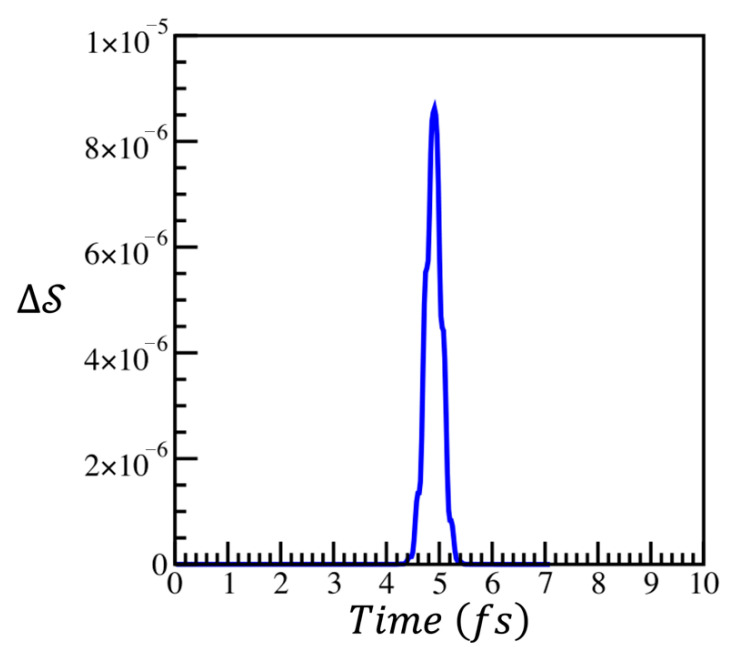
The entropy difference between the compacted, $\rho_{c}^{s}$, and uncompacted, $\rho^{s}$, sudden approximation density matrices.

Figure 3 shows an accurate but approximate proportionality of the elements within each block. Again, using the generic elements of Equations (9) and (10), if the elements of the blocks are related by factors, $b_{1,1}\left(t\right)=x_{m,n}^{b}\;b_{m,n}\left(t\right)$ and $c_{1,1}\left(t\right)=x_{m,n}^{c}\;c_{m,n}\left(t\right)$, these factors are about linearly dependent upon the dipole transition moments of the relevant states. After the pulse the proportionality is exact. This leads directly to the results shown in Figure 4.

The time dependence of each block in the sudden approximation surprisal can be described by one single complex number. It is this accurate approximation which will be exploited in this work to compact the surprisal.

As a preliminary to the discussion on compaction, it is useful to see Equation (8) written out explicitly for the surprisal of the reduced N_2_ system being used in this work,

I=X ΣG+1↔X ΣG+1X ΣG+1↔b Πu1X ΣG+1↔c Πu1X ΣG+1↔e Πu1⋮b Πu1↔b Πu1b Πu1↔c Πu1b Πu1↔e Πu1⋮⋮c Πu1↔c Πu1c Πu1↔e Πu1⋮⋮⋮e Πu1↔e Πu1.

## 6. Compaction in the Sudden Approximation

The $n^{2}$ Gelfand matrices, $\left\{E_{ij}\right\}$, offer a very useful basis set for constructing the surprisal of a density matrix of maximal entropy. This is because at maximal entropy the surprisal is guaranteed to be a linear combination of these Gelfand matrices as constraints,

(11) $$\begin{array}{c} I\left(t\right)=\sum_{i,j=1}^{n,n}\lambda_{i,j}^{E}\left(t\right)E_{ij},\; \end{array}$$

where the indices i and j span all the vibronic states and where the $\lambda_{i,j}^{E}\left(t\right)$ are the corresponding Lagrange multipliers, except for when i=j, the $\lambda_{i,j}^{E}\left(t\right)$ which correspond to the Gelfand matrices will, in general, be complex. Therefore, thanks to the Hermiticity of the surprisal, this uncompacted I will be described by approximately $n^{2}$ real, time-dependent parameters. Hence, the computational complexity of the uncompacted surprisal of an n×n matrix is $O\left(n^{2}\right)$. The objective of the compaction is to construct an exact or approximation of the surprisal, I, with significantly reduced computational complexity. In the specific case of the sudden approximation, we shall show how to reduce it to $O\left(n\right)$.

Begin by defining the sudden approximation surprisal as the sum of complex time-dependent matrices

(12) $$\begin{array}{c} I\left(t\right)\equiv \sum_{i}X_{i}\left(t\right). \end{array}$$

These $\left\{X_{i}\left(t\right)\right\}$ can be put into four groups.

(1) The normalisation group. The first group consists of just one constraint, the normalisation constraint

(13) $$\begin{array}{c} X_{0}\left(t\right)=\lambda_{0}\left(t\right)I. \end{array}$$

The Lagrange multiplier of this normalisation constraint, $\lambda_{0}\left(t\right)$, for the compacted density matrix is calculated from imposing the normalisation at every point in time by the implicit equation: $\mathrm{tr}\left[\rho \left(t\right)\right]=1$.

(2) The diagonal group. The second group consists of the $X_{i}\left(t\right)$ for each diagonal element of I. There will be $v_{tot}$ such constraints. For the reduced N_2_ system, consisting of a ground electronic state with a single vibrational state and three excited electronic states with $v_{j}=8,\;4$ and four vibrational states each, there will be 17 $X_{i}\left(t\right)s$ in the diagonal group. These are listed in Table 2.

The third and fourth groups of $\left\{X_{i}\left(t\right)\right\}$ will take advantage of the surprisal analysis above.

(3) The intra-electronic group. These are the constraints corresponding to the diagonal blocks of Equation (8), the $\left[i↔i\right]$, which contain the information about the coherences between vibrational states within a specific electronic state. The number of intra-electronic constraints will be the number of electronic states. For the reduced N_2_ system used in this work, there will be three $X_{i}\left(t\right)s$ in this group. These are listed in Table 3.
(4) The inter-electronic group. These are the constraints corresponding to the off-diagonal blocks of Equation (8), the $\left[i↔j\right]$ with i≠j, which contain the coherences between the vibrational states of different electronic states. The number of inter-electronic constraints will be the number of possible electronic state transitions. For the N_2_ system, there are six $X_{i}\left(t\right)s$ in this group. These are listed in Table 4.

The $X_{i↔j}^{\prime }$ in Table 3 and Table 4 are time-independent. The actual values of the elements of $X_{i↔j}^{\prime }$ can be defined from the surprisal at any point after the perturbation, $t_{0}<t_{f}$, $I\left(t_{f}\right)=\sum_{i,j=\left\{G,E1,\ldots \right\}}X_{i↔j}^{\prime }$. I has the same structure as Equation (8), and so the $X_{i↔j}^{\prime }$ are sparse matrices that have the same dimensionality as ρ and I, that is, $v_{tot}\times v_{tot}$. The $\left[i↔j\right]$ in Equations (9) and (10), with dimensionality $v_{i}\times v_{j}$, are sub-matrices of these $X_{i↔j}^{\prime }$,

$$X_{i↔j}^{\prime }=\left(\begin{array}{ccc} 0 & 0 & 0\\ 0 & \left[i↔j\right] & 0\\ 0 & 0 & 0 \end{array}\right),$$

where 0s are zero-matrices of varying dimensions.

For one to separate and approximately factorise the time-dependent constraints, $\left\{X_{i}\left(t\right)\right\}$, into time-dependent Lagrange multipliers, $\left\{\lambda_{i}\left(t\right)\right\}$, and time-independent $\left\{X_{i↔j}^{\prime }\right\}$, the individual elements of the $X_{i↔j}^{\prime }$, which effectively means the individual elements of the $\left[i↔j\right]$ blocks, need to be scaled relative to one another.

Using the generic elements in Equation (9), the time-independent coefficients, $x_{m,n}^{b}$, such that $b_{1,1}\left(t\right)=x_{m,n}^{b}\;b_{m,n}\left(t\right)$, can be defined with the values of $b_{1,1}$ and $b_{m,n}$ at a specific point in time after the perturbation is over. $x_{m,n}^{b}=b_{1,1}\left(t_{f}\right)/b_{m,n}\left(t_{f}\right)$. Therefore, one can write that $b_{m,n}\left(t\right)=\left(b_{m,n}\left(t_{f}\right)/b_{1,1}\left(t_{f}\right)\right)b_{1,1}\left(t\right)$. In this way, one can write every element of Equation (9) in terms of $b_{1,1}\left(t\right)$ and a constant number, $b_{m,n}\left(t_{f}\right)/b_{1,1}\left(t_{f}\right)$. In this way, the time dependence can be factorised out of Equation (9)

$$\left[i↔j\right]=b_{1,1}\left(t\right)\left(\begin{array}{cccc} b_{1,1}\left(t_{f}\right)/b_{1,1}\left(t_{f}\right) & b_{1,2}\left(t_{f}\right)/b_{1,1}\left(t_{f}\right) & b_{1,3}\left(t_{f}\right)/b_{1,1}\left(t_{f}\right) & \ldots \\ b_{2,1}\left(t_{f}\right)/b_{1,1}\left(t_{f}\right) & b_{2,2}\left(t_{f}\right)/b_{1,1}\left(t_{f}\right) & b_{2,3}\left(t_{f}\right)/b_{1,1}\left(t_{f}\right) & \ldots \\ b_{3,1}\left(t_{f}\right)/b_{1,1}\left(t_{f}\right) & b_{3,2}\left(t_{f}\right)/b_{1,1}\left(t_{f}\right) & b_{3,3}\left(t_{f}\right)/b_{1,1}\left(t_{f}\right) & \ldots \\ \vdots & \vdots & \vdots & \ddots \end{array}\right).$$

The same argument holds for Equation (10). See also the discussion of Figure 2.

The 27 complex $\left\{X_{i}\left(t\right)\right\}$ from Equation (13) and Table 2, Table 3 and Table 4 can be separated into 36 time dependent real Lagrange parameters, $\left\{\lambda_{j}\left(t\right)\right\}$, and time independent constraints $\left\{Y_{j}\right\}$.

(14) $$\begin{array}{c} \sum_{k=0}^{26}X_{k}\left(t\right)=\sum_{j=0}^{35}\lambda_{j}\left(t\right)Y_{j}\;\; \end{array}$$

This separation is explicitly shown in Table 5. All of the Lagrange multipliers in this table are either purely real or purely imaginary.

The compressed, sudden surprisal is constructed from the constraints and corresponding Lagrange multipliers in Table 5.

(15) $$\begin{array}{c} I_{c}=\sum_{j=0}^{N_{c}}\lambda_{j}\left(t\right)Y_{j}\; \end{array}$$

Equations (11), (12) and (14) can be combined to show clearly the compaction of the reduced N_2_ system

$$\sum_{i,j}^{17,17}\lambda_{i,j}^{E}\left(t\right)E_{i,j}\overset{compaction}{\Rightarrow }\sum_{k=0}^{26}X_{k}\left(t\right)=\sum_{j=0}^{35}\lambda_{j}\left(t\right)Y_{j}.$$

In other words, we reduced the number of constraints from $N=17^{2}$ real time-dependent parameters to $N_{c}=36$ (the intermediate summation is over 36 complex time-dependent parameters). This method will next be used to compress the surprisal of the sudden approximation density matrix of the reduced N_2_ system as it is perturbed by an optical pulse.

The surprisal, $I^{s}=-\ln\left[\rho^{s}\right]$, can be directly propagated using the Liouville–von Neumann equation, $i\partial \ln\left[\rho^{s}\right]/\partial t=\left[H^{s},\ln\left[\rho^{s}\right]\right]$. As before, the $H^{s}$ is the sudden approximation of the Hamiltonian.

Propagating the $\ln\left[\rho^{s}\left(t\right)\right]$ in this way produces an uncompacted sudden approximation surprisal, $I^{s}$. The propagation also produces the $\left\{\lambda_{i,j}^{E}\left(t\right)\right\}$ from which the $\left\{\lambda_{i}\left(t\right)\right\}$ needed for the compression are calculated. The compacted, sudden surprisal, $I_{c}$, can therefore be calculated using Equation (15) with the $\left\{\lambda_{i}\left(t\right)\right\}$ and $\left\{Y_{j}\right\}$ from Table 5. From the compacted, sudden surprisal one can compute the compacted sudden density matrix and thereby its entropy. The normalisation, Equation (13), ensures that the entropy of the compacted density matrix remains constant to within the noise level of <10^−12^. The normalisation of the uncompacted density matrix in the sudden limit is inherently insured by the solution of the Liouville–von Neumann equation with a Hermitian Hamiltonian.

Figure 4 shows the entropy difference, ∆S, calculated using Equation (1), between the compacted and uncompacted sudden approximation density matrices, $\rho^{s}=\exp\left[-I^{s}\right]\;$ and $\rho_{c}^{s}=\exp\left[-I_{c}^{s}\right]$, $\Delta S=tr\left[\rho^{s}\ln\left[\rho_{c}^{s}\right]\right]-tr\left[\rho^{s}\ln\left[\rho^{s}\right]\right]$. As noted above, the entropies of these two densities are constant in time to better than 10^−12^.

Figure 4 shows that, during the time interval of the pulse duration, the compression causes some loss of information from the surprisal. However, after the pulse is finished, the surprisal is compacted lossless.

$$\begin{array}{c} Lossy\\ Lossless \end{array}\;\;\;\;\begin{array}{c} t_{0}-2.3\sigma ≲t≲t_{0}+2.3\sigma \\ all\;other\;t \end{array}$$

Furthermore, the loss of information in the surprisal for the duration of the perturbation is small, $\Delta S<10^{-5}$.

Examination of Table 5 shows that of the 36 constraints needed to fully reproduce the state, 18, *i* = 0 to 17, are “diagonal” ones meaning that they serve to match the diagonal elements of the density matrix, as shown in Table 2. The other 18 constraints are needed because of the entanglement created by the pulse. These are the constraints numbered 18 to 35 in Table 5.

## 7. Conclusions

This work has shown how the structure of the Hamiltonian can be used to compact the dynamics of a perturbed, *entangled* system. The system used in the discussion was the electronic and vibrational states of the N_2_ molecule. When the sudden approximation is valid, the energy differences between the vibrational states of specific electronic states can be ignored. Hence, in the sudden approximation, the different vibrational states of a given electronic state are de facto degenerate. The sudden approximation Hamiltonian therefore only contains information about the energy differences between electronic states.

A technical development that is essential is the recognition and explicit application of a Lie algebra that uses the Gelfand matrices as generators to describe the dynamics. It is made possible by the recognition that almost all computations are carried out in a finite dimensional Hilbert space or Liouville space. This technique enables the use of linear equations of motion.

The sudden approximation creates a structure in the surprisal of the density matrix of maximal entropy, which can be exploited in its compaction. This structure allows one to compact the surprisal from $O\left({v_{tot}}^{2}\right)$ constraints and time-dependent, real Lagrange multipliers, down to $O\left(v_{tot}\right)$. There is a very small loss of information in the compaction for the duration of the pulse. However, after the pulse is finished, the compaction is lossless.

## Figures and Tables

**Table 1:** The energy differences and dipole transition moments between the ground vibrational-electronic state and excited diabatic electronic-vibrational states. Adapted from [29].

Electronic State	Vibrational State, *v*	State Index, *i*	Energy (eV), $E_{i}$	Dipole Transition Moment (a.u.), $\mu_{1,i}$
X ΣG+1	0	1	0	
b Πu1	0	2	12.50	5.20
	1	3	12.58	12.41
	2	4	12.66	18.47
	3	5	12.75	25.14
	4	6	12.84	31.62
	5	7	12.98	7.14
	6	8	13.06	6.63
	7	9	13.16	16.19
c Πu1	0	10	12.91	26.80
	1	11	13.21	26.80
	2	12	13.48	8.77
	3	13	13.74	−
e Πu1	0	14	13.10	2.24
	1	15	13.35	16.97
	2	16	13.58	16.88
	3	17	13.82	16.06

**Table 2:** The diagonal constraints of Equation (12), written explicitly for the reduced N_2_ system.

Electronic State	Vibrational State Quantum Number	
X ΣG+1	0	$X_{1}\left(t\right)=\lambda_{1,1}^{E}\left(t\right)E_{1,1}$
b Πu1	0	$X_{2}\left(t\right)=\lambda_{2,2}^{E}\left(t\right)E_{2,2}$
	1	$X_{3}\left(t\right)=\lambda_{3,3}^{E}\left(t\right)E_{3,3}$
	2	$X_{4}\left(t\right)=\lambda_{4,4}^{E}\left(t\right)E_{4,4}$
	3	$X_{5}\left(t\right)=\lambda_{5,5}^{E}\left(t\right)E_{5,5}$
	4	$X_{6}\left(t\right)=\lambda_{6,6}^{E}\left(t\right)E_{6,6}$
	5	$X_{7}\left(t\right)=\lambda_{7,7}^{E}\left(t\right)E_{7,7}$
	6	$X_{8}\left(t\right)=\lambda_{8,8}^{E}\left(t\right)E_{8,8}$
	7	$X_{9}\left(t\right)=\lambda_{9,9}^{E}\left(t\right)E_{9,9}$
c Πu1	0	$X_{10}\left(t\right)=\lambda_{10,10}^{E}\left(t\right)E_{10,10}$
	1	$X_{11}\left(t\right)=\lambda_{11,11}^{E}\left(t\right)E_{11,11}$
	2	$X_{12}\left(t\right)=\lambda_{12,12}^{E}\left(t\right)E_{12,12}$
	3	$X_{13}\left(t\right)=\lambda_{13,13}^{E}\left(t\right)E_{13,13}$
e Πu1	0	$X_{14}\left(t\right)=\lambda_{14,14}^{E}\left(t\right)E_{14,14}$
	1	$X_{15}\left(t\right)=\lambda_{15,15}^{E}\left(t\right)E_{15,15}$
	2	$X_{16}\left(t\right)=\lambda_{16,16}^{E}\left(t\right)E_{16,16}$
	3	$X_{17}\left(t\right)=\lambda_{17,17}^{E}\left(t\right)E_{17,17}$

**Table 3:** The intra-electronic constraints of Equation (12), written explicitly for the reduced N_2_ system.

b Πu1↔b Πu1	$X_{18}\left(t\right)=Re\left[\lambda_{2,3}^{E}\left(t\right)\right]Re\left[X_{b↔b}^{\prime }\right]+iIm\left[\lambda_{2,3}^{E}\left(t\right)\right]Im\left[X_{b↔b}^{\prime }\right]$
c Πu1↔c Πu1	$X_{19}\left(t\right)=Re\left[\lambda_{10,11}^{E}\left(t\right)\right]Re\left[X_{c↔c}^{\prime }\right]+iIm\left[\lambda_{10,11}^{E}\left(t\right)\right]Im\left[X_{c↔c}^{\prime }\right]$
e Πu1↔e Πu1	$X_{20}\left(t\right)=Re\left[\lambda_{14,15}^{E}\left(t\right)\right]Re\left[X_{e↔e}^{\prime }\right]+iIm\left[\lambda_{14,15}^{E}\left(t\right)\right]Im\left[X_{e↔e}^{\prime }\right]$

**Table 4:** The inter-electronic constraints of Equation (12), written explicitly for the reduced N_2_ system.

X ΣG+1↔b Πu1	$X_{21}\left(t\right)=Re\left[\lambda_{1,2}^{E}\left(t\right)\right]Re\left[X_{X↔b}^{\prime }\right]+iIm\left[\lambda_{1,2}^{E}\left(t\right)\right]Im\left[X_{X↔b}^{\prime }\right]$
X ΣG+1↔c Πu1	$X_{22}\left(t\right)=Re\left[\lambda_{1,10}^{E}\left(t\right)\right]Re\left[X_{X↔c}^{\prime }\right]+iIm\left[\lambda_{1,10}^{E}\left(t\right)\right]Im\left[X_{X↔c}^{\prime }\right]$
X ΣG+1↔e Πu1	$X_{23}\left(t\right)=Re\left[\lambda_{1,14}^{E}\left(t\right)\right]Re\left[X_{X↔e}^{\prime }\right]+iIm\left[\lambda_{1,14}^{E}\left(t\right)\right]Im\left[X_{X↔e}^{\prime }\right]$
b Πu1↔c Πu1	$X_{24}\left(t\right)=Re\left[\lambda_{2,10}^{E}\left(t\right)\right]Re\left[X_{b↔c}^{\prime }\right]+iIm\left[\lambda_{2,10}^{E}\left(t\right)\right]Im\left[X_{b↔c}^{\prime }\right]$
b Πu1↔e Πu1	$X_{25}\left(t\right)=Re\left[\lambda_{2,14}^{E}\left(t\right)\right]Re\left[X_{b↔e}^{\prime }\right]+iIm\left[\lambda_{2,14}^{E}\left(t\right)\right]Im\left[X_{b↔e}^{\prime }\right]$
c Πu1↔e Πu1	$X_{26}\left(t\right)=Re\left[\lambda_{10,14}^{E}\left(t\right)\right]Re\left[X_{c↔e}^{\prime }\right]+iIm\left[\lambda_{10,14}^{E}\left(t\right)\right]Im\left[X_{c↔e}^{\prime }\right]$

**Table 5:** The 27 time-dependent complex constraints, $\left\{X_{i}\left(t\right)\right\}$, from Equation (13) and Table 2, Table 3 and Table 4 are collected and separated into 36 time-dependent real Lagrange parameters, $\left\{\lambda_{i}\left(t\right)\right\}$, and time independent constraints $\left\{Y_{i}\right\}$.

i	$Y_{i}$	$\lambda_{i}(t)$	i	$Y_{i}$	$\lambda_{i}(t)$
0	I	$\ln\left[Z\left(t\right)\right]$	18	$Re\left[X_{b↔b}^{\prime }\right]$	$Re\left[\lambda_{1,2}^{E}\left(t\right)\right]$
1	$E_{1,1}$	$\lambda_{1,1}^{E}\left(t\right)$	19	$Im\left[X_{b↔b}^{\prime }\right]$	$iIm\left[\lambda_{1,2}^{E}\left(t\right)\right]$
2	$E_{2,2}$	$\lambda_{2,2}^{E}\left(t\right)$	20	$Re\left[X_{c↔c}^{\prime }\right]$	$Re\left[\lambda_{1,10}^{E}\left(t\right)\right]$
3	$E_{3,3}$	$\lambda_{3,3}^{E}\left(t\right)$	21	$Im\left[X_{c↔c}^{\prime }\right]$	$iIm\left[\lambda_{1,10}^{E}\left(t\right)\right]$
4	$E_{4,4}$	$\lambda_{4,4}^{E}\left(t\right)$	22	$Re\left[X_{e↔e}^{\prime }\right]$	$Re\left[\lambda_{1,14}^{E}\left(t\right)\right]$
5	$E_{5,5}$	$\lambda_{5,5}^{E}\left(t\right)$	23	$Im\left[X_{e↔e}^{\prime }\right]$	$iIm\left[\lambda_{1,14}^{E}\left(t\right)\right]$
6	$E_{6,6}$	$\lambda_{6,6}^{E}\left(t\right)$	24	$Re\left[X_{X↔b}^{\prime }\right]$	$Re\left[\lambda_{1,2}^{E}\left(t\right)\right]$
7	$E_{7,7}$	$\lambda_{7,7}^{E}\left(t\right)$	25	$Im\left[X_{X↔b}^{\prime }\right]$	$iIm\left[\lambda_{1,2}^{E}\left(t\right)\right]$
8	$E_{8,8}$	$\lambda_{8,8}^{E}\left(t\right)$	26	$Re\left[X_{X↔c}^{\prime }\right]$	$Re\left[\lambda_{1,10}^{E}\left(t\right)\right]$
9	$E_{9,9}$	$\lambda_{9,9}^{E}\left(t\right)$	27	$Im\left[X_{X↔c}^{\prime }\right]$	$iIm\left[\lambda_{1,10}^{E}\left(t\right)\right]$
10	$E_{10,10}$	$\lambda_{10,10}^{E}\left(t\right)$	28	$Re\left[X_{X↔e}^{\prime }\right]$	$Re\left[\lambda_{1,14}^{E}\left(t\right)\right]$
11	$E_{11,11}$	$\lambda_{11,11}^{E}\left(t\right)$	29	$Im\left[X_{X↔e}^{\prime }\right]$	$iIm\left[\lambda_{1,14}^{E}\left(t\right)\right]$
12	$E_{12,12}$	$\lambda_{12,12}^{E}\left(t\right)$	30	$Re\left[X_{b↔c}^{\prime }\right]$	$Re\left[\lambda_{2,10}^{E}\left(t\right)\right]$
13	$E_{13,13}$	$\lambda_{13,13}^{E}\left(t\right)$	31	$Im\left[X_{b↔c}^{\prime }\right]$	$iIm\left[\lambda_{2,10}^{E}\left(t\right)\right]$
14	$E_{14,14}$	$\lambda_{14,14}^{E}\left(t\right)$	32	$Re\left[X_{b↔e}^{\prime }\right]$	$Re\left[\lambda_{2,14}^{E}\left(t\right)\right]$
15	$E_{15,15}$	$\lambda_{15,15}^{E}\left(t\right)$	33	$Im\left[X_{b↔e}^{\prime }\right]$	$iIm\left[\lambda_{2,14}^{E}\left(t\right)\right]$
16	$E_{16,16}$	$\lambda_{16,16}^{E}\left(t\right)$	34	$Re\left[X_{c↔e}^{\prime }\right]$	$Re\left[\lambda_{10,14}^{E}\left(t\right)\right]$
17	$E_{17,17}$	$\lambda_{17,17}^{E}\left(t\right)$	35	$Im\left[X_{c↔e}^{\prime }\right]$	$iIm\left[\lambda_{10,14}^{E}\left(t\right)\right]$

## Data Availability

The raw data supporting the conclusions of this article and the relevant codes will be made available by the authors on request.
